# Readability and Content Assessment of Informed Consent Forms for Phase II-IV Clinical Trials in China

**DOI:** 10.1371/journal.pone.0164251

**Published:** 2016-10-04

**Authors:** Gaiyan Wen, Xinchun Liu, Lihua Huang, Jingxian Shu, Nana Xu, Ruifang Chen, Zhijun Huang, Guoping Yang, Xiaomin Wang, Yuxia Xiang, Yao Lu, Hong Yuan

**Affiliations:** 1 The Ethics Committee, the 3rd Xiangya Hospital of Central South University, Changsha, Hunan, China; 2 Clinical Pharmacology Center, the 3rd Xiangya Hospital of Central South University, Changsha, Hunan, China; 3 Center for Medical Experiments, the 3rd Xiangya Hospital of Central South University, Changsha, Hunan, China; Cardiff University, UNITED KINGDOM

## Abstract

**Purpose:**

To explore the readability and content integrity of informed consent forms (ICFs) used in China and to compare the quality of Chinese local ICFs with that of international ICFs.

**Methods:**

The length, readability and content of 155 consent documents from phase II-IV drug clinical trials from the Third Xiangya Hospital Ethics Committee from November 2009 to January 2015 were evaluated. Reading difficulty was tested using a readability formula adapted for the Chinese language. An ICF checklist containing 27 required elements was successfully constructed to evaluate content integrity. The description of alternatives to participation was assessed. The quality of ICFs from different sponsorships were also compared.

**Results:**

Among the 155 evaluable trials, the ICFs had a median length of 5286 words, corresponding to 7 pages. The median readability score was 4.31 (4.02–4.41), with 63.9% at the 2^nd^ level and 36.1% at the 3^rd^ level. Five of the 27 elements were frequently neglected. The average score for the description of alternatives to participation was 1.06, and 27.7% of the ICFs did not mention any alternatives. Compared with Chinese local ICFs, international ICFs were longer, more readable and contained more of the required elements (*P* < 0.05).

**Conclusion:**

The ICFs used in China were difficult to read for most participants. These forms had poor description of alternatives to participation, and failed to provide a high degree of information disclosure, including an explanation of informed consent, follow-up processing of the data/sample, inclusion/exclusion criteria, double blinding, and unpredictable risks. International ICFs had better readability and content integrity than Chinese local ICFs. More efforts should thus be made to improve the quality of consent documents in China.

## Introduction

With the trend of international globalization, China is playing an important role in conducting clinical trials [1, 2]. More than 800 types of new drugs involving 500,000 Chinese subjects have been evaluated in clinical trials every year. As a cornerstone of ethical healthcare research, an informed consent form (ICF) is a requirement for drug clinical trials to provide protection for participants in these trials [3]. Participants can obtain useful information, weigh benefits and risks, and make an informed and voluntary decision to participate from ICFs. Ideally, a valid ICF must provide all necessary information in a way that is easily understood [4].

Although researchers have emphasized the importance of the standardization and perfection of ICFs, many incomplete or inaccurate facets remain. Numerous studies have shown that most potential participants fail to comprehend ICF content [5–9]. Participants commonly misinterpret the purpose of clinical trials, their benefits and risks, side effects, and the ability to withdraw from participation [6–9]. These deficiencies lead to poor comprehension of ICF information and potentially threaten the safety of subjects. Also, ICFs should be written in simple, easy-to-read language to ensure that participants can fully understand all the information in ICFs [10]. However, as a matter of fact, some researchers have not fully accounted for the age, education level and mental ability of subjects or their representatives [11], which may increase the likelihood of regretting their participation or withdrawing from the study [12].

Some previous studies have evaluated the content and readability of ICFs. In 2003, Paasche-Orlow conducted a large-scale, cross-sectional study using the Flesch-Kincaid readability scale to measure the readability of ICFs from institutional review boards (IRBs) [13]. The results showed that IRBs fall short of their own readability standards and need language improvements. Three separate studies [14–16] developed surveys of the content and readability of ICFs for clinical oncology trials and found that consent documents were too lengthy and complex for participants to read. France, Croatia and New Zealand were facing a similar problem in the poor quality of ICFs [11, 17, 18]. As China is undertaking an increasing number of clinical trials, it is of great importance to clarify the quality of ICFs in China. Notably, ICFs from phase I clinical trials were under the intense scrutiny of IRBs and had higher readability scores [16], as these trials primarily target healthy volunteers and aim to evaluate drug risks. By contrast, the quality of ICFs for phase II-IV clinical trials involving diverse participants is inconsistent.

Our study aimed to explore the readability and content integrity of ICFs for phase II-IV clinical trials in China for the first time and to compare the quality of Chinese local ICFs with that of international ICFs, aiming to provide insights into the deficiencies and misunderstanding of consent documents and provide a sound basis for the improvement of ICFs in China.

## Materials and Methods

### Selection criteria for ICFs

ICFs for phase II-IV drug clinical trials approved by the Third Xiangya Hospital Ethics Committee in Changsha, China, from November 2009 to January 2015 were considered for inclusion in this study. ICFs for phase I clinical trials, bioequivalence researches, pharmacokinetic studies and tolerance studies were excluded. Moreover, this study excluded ICFs for medical apparatuses and techniques and those mainly involving questionnaires. Scanned materials were also excluded because of the unavailability of word counts and readability scores. To avoid duplicative reviews, only the original version of each ICF that was approved by the ethics committee was evaluated.

### Approach to data collection

The following items were collected, including details regarding the phase of the study, administration route, drug classification and sponsor. These documents were classified by sponsor as Chinese local ICFs and international ICFs. The sponsors of international ICFs were multinational pharmaceutical companies, including Bayer HealthCare AG, AstraZeneca Pharmaceutical Co. Ltd, Beijing Novartis Pharma Ltd, GlaxoSmithKline Plc and several Japanese pharmaceutical companies, and the sponsors of Chinese local ICFs were Chinese local pharmaceutical companies.

### Readability assessment

All of the materials evaluated were written in Chinese and available in electronic form. The readability analysis was based on an assessment of the text length, font size, number of pages, presence of a flow chart, use of appropriate sub-headings, and readability score. The number of words, font size and number of pages were determined by Microsoft Word. The presence of a flow chart was checked manually. For the English language, readability formulas such as the Flesch Reading Ease score (FRE) and the Gunning Fog Index (GFI) are based on word complexity and sentence length, but these formulas cannot be directly applied to Chinese because of special characteristics of the language [19, 20]. The readability formula adapted for the Chinese language in 1974 by Yang was used in our study to assess the reading difficulty of the material, which correlates well with comprehension tests [21]. The formula was as follows: Y = 3.5921 + 0.8826X_1_−0.0179X_2_, where Y is a comprehension score, X_1_ is the number of words with a stroke number less than 10 divided by the total number of words, and X_2_ is the number of special characters divided by the total number of words. As no computer software with a Chinese readability formula was available, all calculations were performed manually. According to Fry’s methods [22], three paragraphs (each paragraph included 100 words) were randomly selected near the beginning, middle, and end of the document. The stroke number of every word and the number of special characters in the three paragraphs were then counted. Subsequently, X_1_ was calculated by dividing the number of words with a stroke number less than 10 by 300. X_2_ was calculated by dividing the number of special characters by 300. The comprehension score was evaluated from level 1 to 5, with higher values indicating better readability. The readability levels are reported on a standardized normal distribution of the difficulty of the material but are not tied to the concept of educational level. According to a 2012 Chinese residents' health literacy monitoring report [23], only 8.80% of the residents had an adequate level of health information literacy in China. The overall level of health literacy and education is relatively lower in China than in well-developed countries. For our analysis, the “2^nd^ easy” level is generally associated with a secondary school or higher education level, and the “3^rd^ median” level is associated with a vocational or high school level.

### Content assessment

A content evaluation tool for ICFs was designed according to the World Medical Association Declaration of Helsinki, the international ethical guidelines for biomedical research involving human subjects, the International Conference on Harmonization Clinical Practices Guide (ICH GCP), other regulations and specifications, and the references [24–26]. Composed of 27 items, this tool consists of four sections, including general items, rights of subjects, scientific aspects, and ethical aspects. Two researchers rated all items independently for each ICF, and inconsistent findings were arbitrated by a third party. The researchers had obtained GCP certification and been trained in accordance with unified standards and methods.

### Assessment of alternative descriptions

According to the scoring system for alternative descriptions [27], an ICF scored 0 points if it did not mention any alternatives, 1 point if it mentioned alternatives but contained no description, 2 points if it mentioned and described alternatives, and 3 points if it listed or described alternatives in detail. Similarly, two researchers independently scored all documents and resolved disagreements.

### Data analysis

Statistical analyses were performed with SPSS 19.0 software (SPSS Inc., Chicago, IL, USA). Continuous variables were presented as the median with the range. Skewed data were submitted to the Mann-Whitney U test, and count data were submitted to the chi-square test. Note that *P* values < 0.05 were considered statistically significant.

## Results

### Characteristics of ICFs in China

A total of 334 ICFs from the ethics committee database were obtained. Among these forms, 179 ICFs were excluded from our study for the following reasons: 87 were bioequivalence studies, 35 were pharmacokinetic studies, 24 were medical device clinical trials, 20 were phase I clinical studies, 1 was tolerance research, 4 involved questionnaires, 3 involved medical techniques, and 5 were scanned documents. Finally, 155 ICFs were included in our study (104 Chinese local ICFs and 51 international ICFs), and the characteristics of all ICFs are shown in Table 1.

**Table 1:** Characteristics of informed consent forms (N = 155).

Clinical Trial Parameter	No. Consent Forms	Percentage (%)
Phase of study		
II	49	31.6
III	96	61.9
IV	10	6.5
Administration Route		
oral	79	51.0
intravenous injection	39	25.2
inhalation	7	4.5
external use	12	7.7
hypodermic injection	18	11.6
Drug Classification		
anti-infective drugs	27	17.4
cardiovascular system drugs	20	12.9
digestive system drugs	20	12.9
endocrine system drugs	15	9.7
analgesic, antipyretic analgesic and anti-gout drugs	13	8.4
antineoplastic drugs	12	7.7
respiratory system drugs	10	6.5
central nervous system drugs	9	5.8
gynecology and reproductive system drugs	8	5.2
others	21	13.5
Sponsor		
Chinese local pharmaceutical companies	104	67.1
International multinational pharmaceutical companies	51	32.9

### Readability and content assessment of ICFs used in China

The median number of pages for the ICFs used in China was 7 pages (3–25), with 5286 (1808–21302) words (Table 2). A total of 97.4% of ICFs used appropriate section headings, and only 9.0% of ICFs inserted flow charts explaining how the study would be conducted. The most commonly used font sizes were 12-point font and 10.5-point font. The median readability score was 4.31 (4.02–4.41), with 63.9% at the 2^nd^ level and 36.1% at the 3^rd^ level (Table 3). Of the 27 required elements, 5 elements were frequently neglected (occurrence rate ≤50%), including an explanation of informed consent, follow-up processing of the data/sample, inclusion/exclusion criteria, double blinding, and unpredictable risks (Table 4). None of the ICFs contained all analyzed elements. Specifically, 7.7% of the ICFs included more than 25 elements, 47.1% included 21–24 elements, 37.4% included 16–20 elements, and 7.8% included 15 or fewer elements. The average score for the description of alternatives to participation was 1.06, and 27.7% of ICFs did not mention any alternatives (Table 5).

**Table 2:** Comparison of the length and readability between Chinese local ICFs and international ICFs.

ICF information	Total ICFs (N = 155)	Chinese local ICFs (N = 104)	International ICFs (N = 51)	*P* value
Number of pages	7(3–25)	6(3–13)	14(5–25)	<0.001
Word Count	5286(1808–21302)	4383(1807–13049)	9568(3806–21302)	<0.001
ICF with appropriate section headings, n (%)	151(97.4)	101(97.1)	50(98.0)	1.000
ICF with flow chart, n (%)	14(9.0)	2(1.9)	12(23.5)	<0.001
Font size of the text, n (%)				<0.001
12	92(59.3)	52(50.0)	40(78.4)	
10.5	57(36.8)	49(47.1)	8(15.7)	
Other size	6(3.9)	3(2.9)	3(5.9)	
Readability score	4.31(4.02–4.41)	4.31(4.15–4.41)	4.36(4.02–4.41)	<0.001

Data were presented as the median and range or number and percentage.

**Table 3:** Comparison of the distribution between Chinese local ICFs and international ICFs by comprehension score.

Level	1 easiest	2 easy	3 median	4 hard	5 hardest
Comprehension score	4.7029–5.0000	4.3202–4.7028	3.9827–4.3201	3.6002–3.9828	0.0000–3.6001
Total ICFs	0	99(63.9)	56(36.1)	0	0
Chinese local ICFs (No.%)	0	55(52.9)	49(47.1)	0	0
International ICFs (No.%)	0	44(86.3)	7(13.7)	0	0

**Table 4:** Comparison of the content between Chinese local ICFs and international ICFs.

ICF elements, n (%)	Total ICFs (N = 155)	Chinese local ICFs (N = 104)	International ICFs (N = 51)	*P* value
**General items**				
a statement that this is research	116(74.8)	77(74.0)	39(76.5)	0.743
an explanation of informed consent	64(41.3)	35(33.7)	29(56.9)	0.006
confidentiality of records	153(98.7)	102(98.1)	51(100.0)	1.000
who can access the data	146(94.2)	95(91.3)	51(100.0)	0.031
follow-up processing of the data/sample	52(33.5)	23(22.1)	29(56.9)	<0.001
research contact person(s)	152(98.1)	101(97.1)	51(100.0)	0.551
ethics committee contact information	80(51.6)	48(46.2)	32(62.7)	0.052
**Rights of the subject**				
right to refuse	155(100.0)	104(100.0)	51(100.0)	1.000
right to withdraw	114(73.5)	74(71.2)	40(78.4)	0.334
replacement therapy	112(72.3)	69(66.3)	43(84.3)	0.019
right to receive new relevant information	112(72.3)	65(62.5)	47(92.2)	<0.001
**Scientific aspects**				
purpose of the study	153(98.7)	102(98.1)	51(100.0)	1.000
inclusion/exclusion criteria	35(22.6)	28(26.9)	7(13.7)	0.065
number of subjects required	148(95.5)	97(93.3)	51(100.0)	0.096
trial treatment	123(79.4)	74(71.2)	49(96.1)	<0.001
trial procedures	123(79.4)	79(76.0)	44(86.3)	0.136
duration of the subject’s participation	138(89.0)	87(83.7)	51(100.0)	0.002
an explanation of the compared, control or placebo group	111(71.6)	67(64.4)	44(86.3)	0.005
randomized allocation	136(87.7)	92(88.5)	44(86.3)	0.696
double blinding	36(23.2)	14(13.5)	22(43.1)	<0.001
pregnancy test for female subjects	116(74.8)	75(72.1)	41(80.4)	0.265
contraception statement	122(78.7)	79(76.0)	43(84.3)	0.233
**Ethical aspects**				
foreseeable risks	143(92.3)	94(90.4)	49(96.1)	0.339
unpredictable risks	71(45.8)	36(34.6)	35(68.6)	<0.001
individual benefit	147(94.8)	101(97.1)	46(90.2)	0.116
social benefit	81(52.3)	39(37.5)	42(82.4)	<0.001
compensation for injury	146(94.2)	95(91.3)	51(100.0)	0.031

**Table 5:** Comparison of the description of alternatives to participation between Chinese local ICFs and international ICFs.

ICF information	Total ICFs(N = 155)	Chinese local ICFs (N = 104)	International ICFs (N = 51)
Average score	1.06	0.85	1.51
Score, n (%)*			
0	43(27.7)	35(33.7)	8(15.7)
1	68(43.9)	52(50.0)	16(31.4)
2	35(22.6)	15(14.4)	20(39.2)
3	9(5.8)	2(1.9)	7(13.7)

*P<0.001, Chinese local ICFs vs. International ICFs.

### Comparison between Chinese local ICFs and international ICFs

Comparisons of the readability, content, and description of alternatives to participation between Chinese local ICFs and international ICFs are shown in Tables 2–5. Chinese local ICFs were significantly shorter than international ICFs (*P* < 0.001). Most Chinese local and international ICFs used subtitles to clarify details (97.1% vs. 98.0%, respectively, *P* > 0.05). International ICFs were more likely to use illustrations than Chinese local ICFs (23.5% vs. 1.9%, respectively, *P* < 0.001). A significant font size difference between the Chinese local ICFs and international ICFs was found. In addition, the readability score for international ICFs was higher than that for Chinese local ICFs (*P* < 0.001). A total of 86.3% of international ICFs and 52.9% of Chinese local ICFs were at “2^nd^ easy” level (Table 3). Compared with Chinese local ICFs (most contained 18–21 elements), international ICFs (most contained 22–25 elements) had more of the required items. The average score for the description of alternatives was 1.51 for international ICFs, which was higher than that for Chinese local ICFs (Table 5).

## Discussion

To the best of our knowledge, the current study was the first to use a readability formula and a content evaluation tool for the readability and content analysis of ICFs used in China. This initial attempt allowed us to explore the defects of these ICFs in detail and to explore the differences between Chinese local and international ICFs. The content evaluation tool included comprehensive information and considered the extent to which the forms contained key information that was useful for subjects to make decisions. Each ICF was rated by two researchers independently, thereby avoiding rater bias.

The results showed that the median readability score of the evaluated ICFs was 4.31 at a 3^rd^ level, with 63.9% at the 2^nd^ level and 36.1% at the 3^rd^ level. The education level of Chinese resident was relatively low, with 37.46% at or below the primary school level and 36.18% at the secondary school level [23], leading to the reading difficulties for the participants. Similarly, other studies found that ICFs from the United States, New Zealand, France, Spain, and Croatia failed to offer subjects complete information using clear and direct language [11, 16–18, 28, 29]. 97% of Canadian ICFs used appropriate section headings to label the separate sections of content, and such headings were helpful for reading comprehension, and the results are just consistent with ours [16]. Additionally, multinational pharmaceutical companies usually included flow charts visually depicting how the study would be conducted and markedly drawing attention to the materials to improve comprehension among participants with low literacy levels [30, 31]. Our results indicated that 12-point font and 10.5-point font were the most common font sizes of Chinese ICFs, whereas 11-point font was typically used in English ICFs [16], indicating that larger fonts improve the understanding of ICFs especially for the elderly subjects and those with poor eyesight.

Compared with Chinese local ICFs, international ICFs had better readability, contained more detailed and complete content. Generally, people are not willing to spend much time reading consent forms, and they are likely to read no more than 4 pages of an ICF [32, 33], but the median number of pages for the international ICFs was 14, which was beyond the accepted range of normal. Strategies for reducing the length of ICFs while concisely retaining all essential elements are worth exploring in both Chinese local ICFs and international ICFs. Among the ICFs reviewed, we found that the majority did not perform well according to the content evaluation tool. Our results found that ICFs used in China did not provide full information disclosure, and 7.8% of the ICFs contained 15 or fewer elements. Detailed explanations of these required items could help subjects to weigh the advantages and disadvantages and better safeguard their interests [34]. Incredibly, ICFs used in China attached great importance to the contact information of the researcher but not to the contact information of the ethics committee. Chinese local studies have promised direct benefits to participants but have neglected the description of social benefits, which prevents participants from realizing the social value of clinical research, such as the promotion of medical developments. Only 45.8% of the ICFs used in China emphasized the unpredictable risks of clinical trials. Researchers may intend to minimize patient concerns by omitting these serious risks, but this is considered unacceptable [16]. No unified template or sample text is provided by law, in ordinances or in guidelines to clearly define all the necessary elements of ICFs may lead to the poor quality of Chinese ICFs.

Based on the results from our study, ethics committees and other reviewing institutions should establish standards for the length, readability and content of consent documents. ICFs should theoretically be limited to no more than 4200 characters (approximately 6 pages). According to the findings by Liao [35], a subject can read Chinese at a rate of 562 to 622 words per minute, and the average person would likely read the document in 5 to 7 minutes [15]; thus, limiting the length of ICFs to 4200 characters is reasonable. However, in a real-world situation, restricting ICFs to 6 pages may assume the risk of presenting incomplete information to participants in complex trials; thus, fewer words should be used to state more information in order to be concise and succinct in ICFs. Ethics committees should develop Chinese readability software. Every consent document should be checked by the same software prior to submission to ensure that ICFs are at or below the 2^nd^ level. We encourage the use of section headings and illustrations, which improve comprehension for participants. To ensure content integrity, ethics committees should offer templates and sample text to investigators, and these documents must be written in clear and direct language to ensure that subjects understand them accurately. Moreover, we should improve the process of ethical review by implementing consistent training practices for consenters.

Several limitations of this study must be considered. First, our sample was relatively small and all the ICFs were obtained from one single ethics committee. Although all ICFs were sampled randomly to ensure representativeness, it was difficult to ensure complete consistency among the ICFs used in China. Further study should be carried out to assess ICFs from multiple ethics committees. Second, compared with the FRG and GFI, which have been used to assess English readability for many years, the Chinese readability formula has not been generalized and applied widely, even though it was invented in 1974. We were unable to use a computer program with readability formulas adapted for the Chinese language, and the manual review method increased the likelihood of random error. Third, we did not assessed readability separately for each section, including general items, rights of subjects, scientific aspects, and ethical aspects. Only the first draft of ICFs was included in the analysis which may have caused selective bias. Fourth, the content measurement may not accurately reflect the perspectives of patients who read these ICFs.

## Conclusion

The ICFs used in China were difficult to read for most participants. And these documents had poor description of alternatives to participation, and failed to provide a high degree of information disclosure, including an explanation of informed consent, follow-up processing of the data/sample, inclusion/exclusion criteria, double blinding, and unpredictable risks. International ICFs had better readability and content integrity than Chinese local ICFs. Greater efforts should be devoted to improving the readability and content of written documents to ensure the rights of potential participants.
